# Remaining useful life prognosis of turbofan engines based on deep feature extraction and fusion

**DOI:** 10.1038/s41598-022-10191-2

**Published:** 2022-04-20

**Authors:** Cheng Peng, Yufeng Chen, Weihua Gui, Zhaohui Tang, Changyun Li

**Affiliations:** 1 Hunan University of Technology, Zhuzhou, 412007 China; 2 Central South University, Changsha, 410083 China

**Keywords:** Mechanical engineering, Information technology

## Abstract

In turbofan engine datasets, to address problems, such as noise interference, diverse data types, large data volumes, complex feature extraction, inability to effectively describe degradation trends, and poor remaining useful life (RUL) prognosis effects, a remaining useful life prognosis model combining an improved stack sparse autoencoder (imSSAE) and an improved echo state network (imESN) is proposed in this paper. First, the 3-sigma criterion is adopted to remove the noise and reconstruct the data, and then the deep features of the engine are extracted by using an imSSAE and fused into health indicator (HI) curves describing the engine degradation trend. Finally, an attention mechanism is introduced into an imESN to adaptively process different types of data and obtain the RUL. The experimental results based on the Commercial Modular Aero-Propulsion System Simulation (C-MAPSS) dataset show that compared with the other popular RUL prediction models, the combined model proposed in this paper has higher prediction accuracy, and the evaluation indices also show the effectiveness and superiority of the model.

## Introduction

Turbofan engines are the core of an aircraft, and the health status analysis of turbofan engines is very important for aircraft evaluation, safe use and formulating maintenance strategies, yet the RUL is the key index to measure the health status of turbofan engines. Accurately predicting the remaining useful life can not only avoid safety accidents caused by untimely maintenance but also reduce the expensive cost of excessive maintenance^1^. However, due to the numerous measuring points, complex working conditions and numerous data, the RUL prediction of turbofan engines faces great challenges in feature extraction and prediction accuracy. First, the selection of feature extraction methods or models directly determines the effectiveness of features. Second, the health indicators (HIs) reflecting the degradation of turbofan engines determine the prediction accuracy. Scholars at home and abroad have conducted much relevant research in this direction.

Currently, there are two main types of RUL prediction methods: model-based prediction^2^ and data-driven prediction^3^. Model-based RUL prediction establishes a mathematical or physical model to analyze and describe the degradation process of equipment and components. Cao et al.^4^ predicted the RUL of gears by establishing a finite element model and damage propagation model to simulate the crack propagation of gears. Cai et al.^5^ applied the nonlinear Wiener process to build a double nonlinear implicit degradation model and gradually updated the random coefficient and the postdistribution of the actual degradation state to realize RUL prediction; these studies verify the effectiveness of the method.

However, turbofan engines are large, precise and complex internal structure equipment. The working environment, manufacturing materials and other uncertain factors of this kind of equipment make it difficult to establish an accurate model; consequently, the above method has certain limitations. Therefore, the data-driven method has become the mainstream of RUL prediction^6^. Zhao et al.^7^ established a fusion model of aero-engine monitoring information based on Euclidean distance and a degradation model based on the nonlinear drift Wiener process to estimate the RUL of the engine. Berghout et al.^8^ proposed a data-driven scheme based on the algorithm of an online sequence limit learning machine to predict the RUL; this scheme enhanced the feature representation by using a laminated self-encoder, introduced a dynamic forgetting function to improve the dynamic tracking ability of data, and developed a new update selection strategy. The effectiveness and accuracy of the prediction model were verified in turbofan engine experiments.

Additionally, with the development of deep learning technology, some deep learning models have been applied to research RUL prognosis based on data-driven methods. Although the deep learning model can mine effective features, supervised learning methods, such as convolutional neural networks, need many labeled data for fine-tuning; actually, labeled data are difficult to obtain. Autoencoders (AEs) are typical unsupervised network models, which can extract the deep expression of unlabeled input data by unsupervised learning. The main forms of AEs include noise reduction AEs, sparse AEs, stack AEs and variational AEs. Because both feature extraction and nonlinear dimensionality reduction are important functions, AEs are widely used in RUL prediction of rolling bearings, turbofan engines and other components. Xia et al.^9^ classified the collected bearing signals into different degradation stages by using an AE and he established a regression model by using a shallow neural network for each stage; the experiment confirmed the effectiveness of this method for simple signal prediction. Ma et al.^10^ applied the stacked sparse AE to automatically extract the performance degradation characteristics of multiple sensor datasets of turbofan engines, fused the features through multilayer self-learning, and finally predicted the RUL by using logistic regression.

The above research shows that the sparse AE can extract proper features under bad conditions and reduce the dimension to a sparse expression, while the stacked encoder can carry out layer-by-layer unsupervised pretraining. Combining the advantages of the two models, we apply the sparse AE to extract the deep features of the sensor dataset and then stack the sparse AE to form a new model called the improved stack sparse autoencoder (imSSAE), which will reduce the multidimensional deep features to one-dimensional features. This structure not only reduces the feature dimension but also ensures the effectiveness of the feature and simplifies the feature processing in the subsequent neural network.

In addition to extracting features, RUL prediction also needs to construct HIs. Many examples show that the quality of an HI greatly affects the effectiveness of prediction methods, so it is necessary to construct an effective HI. Yu et al.^11^ obtained the one-dimensional operating HI value through the automatic encoder of a bidirectional recurrent neural network and then used similarity curve matching technology to estimate the RUL. Wu et al.^12^ created a multiscale convolutional neural network (MSCNN) with a frequency signal as input and then combined the network with the "bathtub curve" and inverse hyperbolic tangent function to construct an HI.

RUL prediction is the final step of the study. By examining many studies, we found that the echo state network (ESN) is a new type of recurrent neural network (RNN) that can randomly deploy large-scale coefficient-linked neurons to form the network’s hidden layer, which is generally called a "reservoir", and simulate the time dynamics information of complex sequences. In addition, the connection between the neurons of the ESN is sparse, and the connection relationship is generated randomly. During network training, the ESN can ensure the global optimization of weights, overcome the local optimization, and has high generalization ability.

The ESN has been preliminarily applied in time series prediction fields, such as life prediction. Zhong et al.^13^ fused the output of a dual reservoir echo state network (DRESN) and then optimized the parameters of the DRESN by using a genetic algorithm (GA). Turbofan engine experiments verified the accuracy and stability of the model. Marco et al.^14^ aggregate the results according to the memory capacity of the ESN. During the mean variance estimation (MVE), an additional ESN is used to estimate the uncertainty of the RUL. Experiments show that the method is superior to static integration and standard MVE in uncertainty estimation tests. Hua et al.^15^ established an ESN structure with multiple inputs and multiple outputs to improve the accuracy of RUL prediction. Parameters such as voltage, current, temperature and reactant pressure are used to predict the RUL. We find that the input layer of the traditional ESN network cannot process different elements at the same time. In this paper, the HI curve value is used as the input of the improved echo state network (imESN). The imESN can adaptively process different input values and carry out RUL prediction of turbofan engines by optimizing network parameters to accelerate convergence and solve the local optimization problem.

In summary, to solve the problems that exist in turbofan engine RUL prognosis, the main contributions of this paper are as follows: (1) the imSSAE is designed to extract and reduce the deep features of the preprocessed data and the corresponding HI curve of each engine is constructed. (2) The RUL value corresponding to each HI curve of the engine unit is predicted by the imESN. The method proposed in this paper outperforms other methods in the HI curve effect, RUL prediction accuracy and error control, thereby proving the feasibility and effectiveness of our method.

The rest of the paper is organized as follows: “Methodologies” presents the basic theory and mainly introduces the model structure of the sparse autoencoder and echo state network. The third section is the focus of this article; this section mainly presents the proposed methodology, structure of the imSSAE and imESN, algorithm, training process, and implementation flow. The fourth section presents the experiment and result analysis, and the final section presents the conclusion and prospect of our model.

## Methodologies

### A. The framework of the Proposed RUL Prognosis model

**Figure 1:**
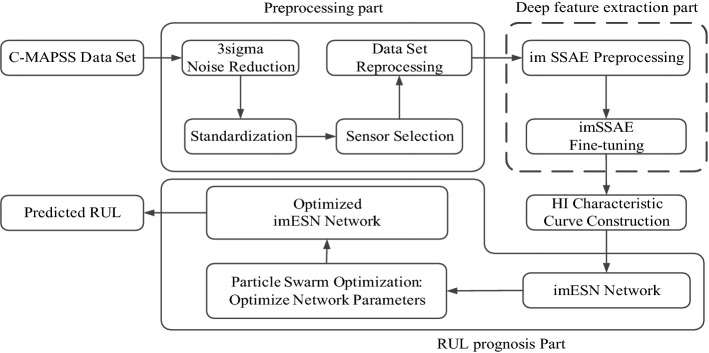
The framework of the proposed model.

The model proposed in this paper combines the advantages of the SSAE in feature extraction and the advantages of the ESN in processing time series data. First, the SSAE is improved to obtain the features and eigenvalues of each engine, then the HI of each engine is constructed by the eigenvalues from different engines in the dataset, and the HI is put into the improved ESN. The curve of HI changes with time; these changes can be considered as a series of continuous eigenvalues. Finally, these eigenvalues are processed by the improved ESN and form the corresponding RUL value. The framework of the entire proposed model is shown in Fig. 1. The model is divided into three main parts, namely, preprocessing, deep feature extraction, and RUL prognosis.

### B. imSSAE

The traditional SSAE trains the whole network layer by layer; the SSAE first pretrains the sparse automatic coder units of each hidden layer unsupervised, and then stacks them, and finally carries out the overall reverse optimization training to obtain a stacked sparse automatic coder structure with multiple hidden layers. This structure can improve training efficiency but insufficiently. In this paper, the SAE (sparse self-encoder), the constituent unit of the SSAE, is improved (the improved SSAE is named imSSAE) to improve the effect of deep feature processing. The following is the reasoning process of the imSSAE. First, the encoding and decoding process of the SAE can be expressed as follows:

1 $$ H = S_{encoder} (b_{1} + W_{1} X) \, $$

2 $$ \hat{X} = S_{decoder} (b_{2} + W_{2} H) $$

where $S\_encoder$ is the encoding activation function;$S\_decoder$ is the decoding activation function; H is the activation value of the hidden layer, X is the input sample data, and $\widehat{\mathrm{X}}$ is the output.$b_{1} ,b_{2}$ are the offsets of the encoding and decoding parts, respectively; and $W_{1} ,W_{2}$ are the weights of the encoding and decoding parts, respectively. The sparse auto encoder, like the auto encoder, applies back propagation and gradient descent algorithms to update and iteratively optimize the weight and offset in the encoder; then, the mean square error function is minimized. The function is expressed in Eq. (3), where $h( \cdot )$ is a hidden layer function, which approximates an identity function and can also make the input close to the output; The goal of the auto encoder is to make the learning function satisfy $h(\mathrm{X})\approx \widehat{\mathrm{X}}$, that is, the output $\widehat{\mathrm{X}}$ is approximately equal to the input X and where $(x^{(i)} ,y^{(i)} )$ corresponds to the input and output, respectively, of the $i - th$ sample.

3 $$ J_{MSE} (W_{1} ,b_{1} ,W_{2} ,b_{2} ) = \frac{1}{n}\sum\limits_{i = 1}^{n} {(\frac{1}{2}||h_{{W_{1} ,b_{1} ,W_{2} ,b_{2} }} (x^{(i)} ) - y^{(i)} ||^{2} )} $$

In fact, the setting of the activation function will affect the result of the hidden layer, the output layer, and the mean square error function. The activation functions generally adopt the *sigmoid* function. When the input value of neurons is far from the zero point, the derivative value of $sigmoid$ will become very small, almost zero. For the turbofan engine dataset, an excessively large number of samples will result in a slow convergence of the model and the problem of gradient disappearance. To solve the above problem, we replace the $sigmoid$ function with the $Tanh$ function in the encoding and decoding parts. the $Tanh$ function converts the input value between $- 1$ and $1$; the derivative ranges from $0$ to $1$, rather than the range of $sigmoid$ from $0$ to $1/4$; and the mean of $Tanh$ is $0$. To a certain extent, compared with $sigmoid$, $Tanh$ delays the period of saturation. In addition, $Tanh$ works better when features are distinctly different; therefore, the $Tanh$ function is applied as the activation function of each sparse auto encoder used in this paper.

During network training, the distribution of input values will be biased as the depth increases and will easily result in gradient disappearance during back propagation. To avoid gradient disappearance, a batch normalization (BN) layer is added in front of each hidden layer of the SSAE so that the encoder forms an "input layer-BN layer-hidden layer" structure. After adding the BN layer, for each neuron of the hidden layer, the input distribution gradually approaches the limit saturation area of the value range after mapping to the nonlinear function and is forcibly pulled back to the standard normal distribution with a mean of 0 and a variance of 1, thus making the input of the nonlinear transformation function fall into the sensitive area, accelerating model learning and simplifying the network parameter adjustment. The processing formula of the BN layer is as follows:

4 $$ \left\{ {\begin{array}{*{20}c} {\mu = \frac{1}{n}\sum\limits_{i = 1}^{n} {x_{l}^{i} } } \\ {\sigma^{2} = \frac{1}{n}\sum\limits_{i = 1}^{n} {(x_{l}^{i} - \mu )^{2} } } \\ \end{array} } \right. $$

5 $$ \left\{ {\begin{array}{*{20}c} {\hat{x}_{l}^{i} = \frac{{x_{l}^{i} - \mu }}{{\sqrt {\sigma^{2} + \varepsilon } }}} \\ {BN(\hat{x}_{l}^{i} ) = \gamma \hat{x}_{l}^{i} + \beta } \\ \end{array} } \right. $$

where $x_{l}^{i}$ is the $i{ - }th$ input data of the $l{ - }th$ layer; $i = 1,2, \ldots ,n$ refers to the number of data passed from the previous layer to the next layer; $\mu$ is the mean of the input data; $\sigma^{2}$ is the variance of the input data; $\hat{x}_{l}^{i}$ is the normalized value of the data; $\varepsilon$ is a small constant term; $\gamma$ and $\beta$ are the training parameters of the network; and BN is a new value through the linear transformation of $\gamma$ and $\beta$ after standardization.

**Figure 2:**
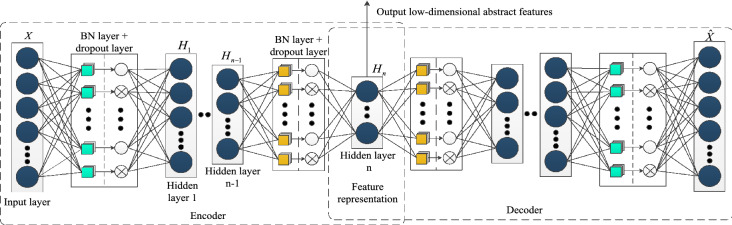
The structure of the imSSAE.

In addition to the problem of gradient disappearance, the traditional SAE adds KL dispersion as a regular term to the loss function to constrain the sparseness of the entire network. However, the turbofan engine dataset studied in this paper is preprocessed, and the deep effective features of the dataset are extracted. Then, normalization limits the deep effective features and the subsequent HI to a range between [0, 1]. The KL dispersion as the sparse constraint of the SAE usually applies to the classification problem with a true value of 0 or 1. Therefore, the average activity $\hat{\rho }_{j}$ cannot be used as a basis to punish the network. To solve this problem, this paper adds a dropout layer to realize the sparsity of the SAE. Similar to the BN layer, the dropout layer is introduced before the activation function during the encoding and decoding of the hidden layer. The dropout mechanism can randomly inactivate the neuron activation value during encoding and decoding (i.e., set a certain probability *p* to 0) to achieve the sparsity constraint, which can be written as:

6 $$ I^{\prime} = {\text{Bernoulli(1,1 - p)}} \cdot \frac{1}{1 - p} \cdot I $$

where $I$ is the input of the original activation function, $I^{\prime}$ is the input of the activation function processed by the dropout layer, and ${\text{Bernoulli(}} \cdot {)}$ represents the Bernoulli distribution. When the activation value is set to 0, the weight and offset of the corresponding neuron have not been updated in learning, and the neuron does not affect the original encoding and decoding process. The structure of the imSSAE is shown in Fig. 2.

### C. HI construction

Currently, most RUL prediction models use the objective function to mark the real RUL value, that is, by inputting the sensor data into the neural network to directly predict the RUL, thus leading to different RUL values for the same health level under the objective function. To solve this problem, this paper adopts engine features to construct an HI curve to describe the degradation trend of the engine. Specifically, after feature extraction by the imSSAE, a set of one-dimensional eigenvalues of each engine is obtained, and each eigenvalue represents the multisensor data information in the engine life cycle, which can also distinguish the fault and degradation state in the data. During the data training, according to the one-dimensional eigenvalues, the real-time state features, historical degradation features and engine failure features in the engine life cycle are obtained. The engine HI constructed in this paper is shown in the following.

7 $$ HI = \sqrt {\sum\limits_{t = 1}^{T} {(F_{t} - F_{end} )^{2} } } \, $$

8 $$ HI^{\prime} = \frac{{HI - HI_{\max } }}{{HI_{\max } - HI_{\min } }} \, $$

where $T$ is the length of the HI curve, $F_{t}$ is the real-time feature of the $t - th$ life cycle, and $F_{end}$ is the failure feature of the engine. During construction, an HI value of 0 indicates that the engine has completely failed, and an HI value of 1 indicates that the engine is normal. The HI degradation state is updated and limited to [0, 1] by Eq. (8). $HI$ and $HI^{\prime}$ are the HI values before and after the update, respectively; and $HI_{\max }$ and $HI_{\min }$ are the maximum and minimum HI values, respectively.

To quantitatively evaluate the effect of the HI curve, time relevance and monotonicity are proposed as evaluation indices in this paper. The time correlation of the HI curve of the $i{ - }th$ engine unit is shown in Eq. (9).

9 $$ Corr_{i} = \frac{{\left| {\sum\limits_{t = 1}^{T} {(HI_{ti} - \overline{{HI_{i} }} )(l_{ti} - \overline{{l_{i} }} )} } \right|}}{{\sqrt {\sum\limits_{t = 1}^{T} {(HI_{ti} - \overline{{HI_{i} }} )^{2} } \sum\limits_{t = 1}^{T} {(l_{ti} - \overline{{l_{i} }} )^{2} } } }} $$

where $T$ is the length of the HI curve, $HI_{ti}$ represents the health value of the $i - th$ engine at the $t - th$ cycle $(i = 1,2, \ldots ,N)$ in the HI curve, $\overline{{HI_{l} }}$ is the average at each cycle in the HI curve, $l_{ti}$ is the $i - th$ cycle of the turbine engine, and $\overline{{l_{l} }}$ is the average of the cycle.

The monotonicity of the HI curve of the $i - th$ engine unit is shown in Eq. (10):

10 $$ Mon_{i} = \left| {\frac{{(Num{\text{ of dQ}}_{i} > 0) - (Num{\text{ of dQ}}_{i} < 0)}}{T - 1}} \right| $$

where ${\text{dQ}}_{i}$ is the derivative of the sequence value in the $i - th$ engine HI curve, $Num{\text{ of dQ}}_{i} > 0$ represents the number of ${\text{dQ}}_{i}$ values greater than 0, and $Num{\text{ of dQ}}_{i} < 0$ represents the number of ${\text{dQ}}_{i}$ values less than 0.

### D. imESN

**Figure 3:**
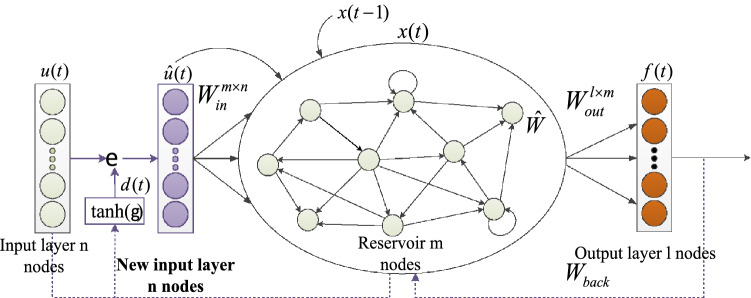
The structure of the imESN.

The traditional ESN network regards the elements of the input layer as an entirety of the same type, but in fact, the input of the ESN network in most cases consists of different elements representing different features. Taking the dataset of the turbofan engine studied in this paper as an example, the input of the neural network is the engine's HI curve value, which essentially represents the different features of the engine. Therefore, we improved the traditional ESN. The attention mechanism is added to the ESN network in this paper to adaptively process the various features of the engine we extracted and to ensure that the elements are completely input into the neural network and that the correct result is outputted. The attention mechanism is defined in Eq. (11):

11 $$ d(t) = \varphi (W_{in} u(t) + \hat{W}x(t - 1) + b_{d} ) $$

where the output $d(t)$ of the attention mechanism is a vector whose dimension is consistent with the input layer state $u(t)$ at time $t$; $\varphi ( \cdot )$ is the activation function, and the activation function in this paper is *tanh*; $W_{in}$ is the connection weight between the input layer and the reservoir; $\hat{W}$ is the state feedback weight of the reservoir; and $b_{d}$ is the offset of the attention mechanism. The previous output state $x(t - 1)$ of the reservoir and the input layer state $u(t)$ at time $t$ are used to determine the importance of each feature of the input layer. After the attention mechanism is updated, the original input becomes Eq. (12).

12 $$ \hat{u}(t) = u(t) \odot d(t) $$

where $\hat{u}(t)$ is the state of the new input and $\odot$ refers to elemental multiplication. When the new input replaces the original input, the state of the imESN reservoir will also change and will be updated, as shown in Eq. (13)

13 $$ \begin{aligned}  x(t) &= (1 - \lambda )\tanh (W_{in} \hat{u}(t) + \hat{W}x(t - 1) \hfill \\ & \quad + W_{back} \hat{f}(t - 1) + \eta ) + \lambda x(t - 1) \hfill \\ \end{aligned} $$

where $x(t)$ is the state of the reservoir at time $t$, $\lambda$ is the leakage rate with the range of ${[0,1]}$, $W_{back}$ is the weight matrix of the input and output feedback, $\hat{f}(t - 1)$ is the state of the output layer at time $(t - 1)$, and $\eta$ is the regularization coefficient. The structure of the imESN is shown in Fig. 3.

To improve the prediction accuracy of the imESN, the internal parameters of the imESN need to be optimized to make the model achieve the best status. The improved particle swarm optimization algorithm is adopted to optimize the parameters of the imESN, as shown in Algorithm 1.

**Figure:**
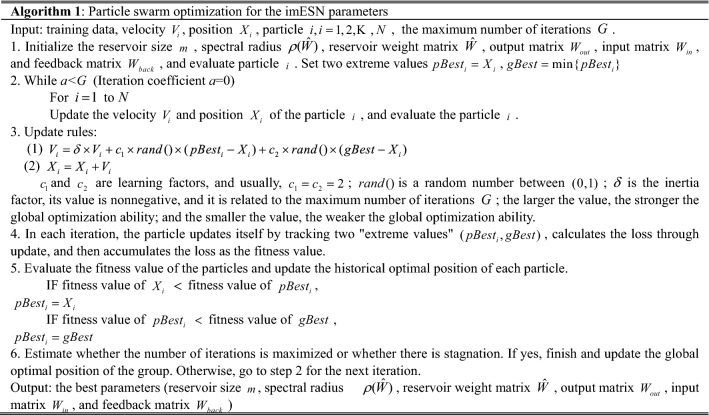

### E. Model training

The goal of training is to minimize the cost function and loss function and to obtain the best parameters. The training of the model in this paper is divided mainly into training the imSSAE and training the imESN. In the fine-tuning stage of the imSSAE, the Adam algorithm^16^ is employed to optimize the parameters. The whole training process adopts early stopping^17^ technology, which can verify the effect of the model during training and avoid overfitting. When the early stopping conditions are met, the final parameters of the model can be selected. The training algorithms of the imSSAE and imESN are as follows.

**Figure:**
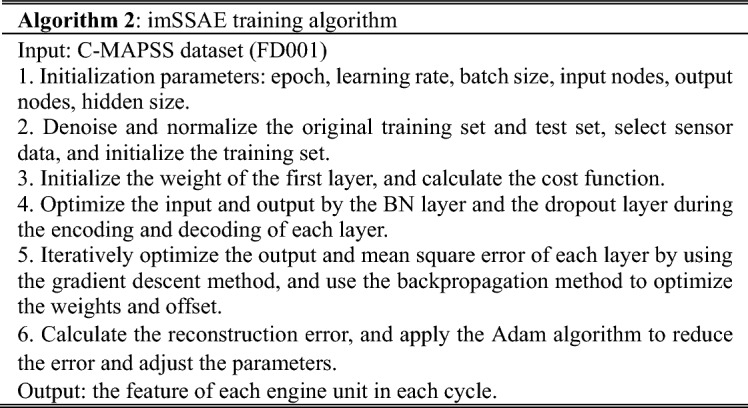

**Figure:**
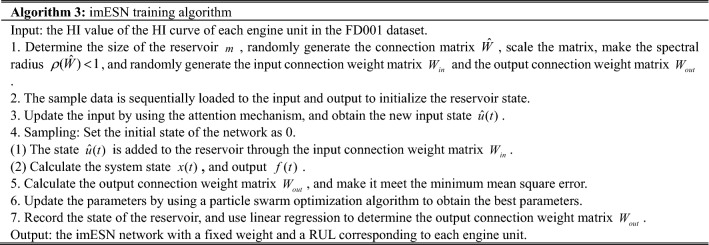

## Experiment and analysis

### A. Dataset description

**Figure 4:**
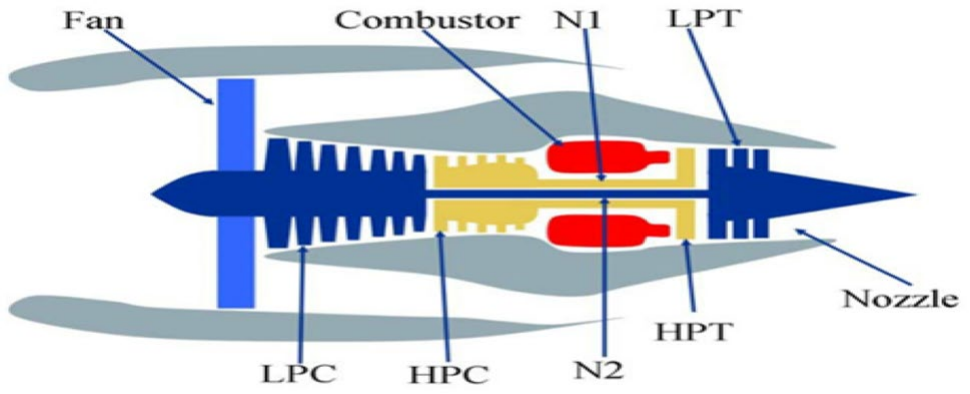
The turbofan engine model.

To verify the effectiveness of the proposed method, this paper adopts the prognostics and health management dataset named C-MAPSS, created by NASA^18^. This dataset includes time series measurements of various pressures, temperatures and speeds of turbofan engines. The engine model is shown in Fig. 4.

The C-MAPSS dataset is a widely used benchmark dataset in RUL prediction; the dataset includes 4 sets of monitoring data (FD001–FD004) under different running conditions of the engine. Each group of monitoring data contains a training set, a test set and an RUL set. Each engine in this dataset has different degrees of initial wear, which is unavailable to users, and much random noise is mixed in the dataset. It can be observed that the engine may fail, and the operating data of the engine stops over time, at this moment, the sensor captures the degradation. There are 21 sensors (such as the total temperature sensor of the fan inlet), 3 running parameters (i.e., flight altitude, Mach number, and throttle resolver angle), engine identification and cycles of each engine. The sensors can capture the engine performance degradation; this ability is the key to realizing RUL prediction. The specific description of the sensors is shown in Table 1.

**Table 1:** Data description of turbofan engine sensors.

Sensor number	Sensor description	Units
1	Fan inlet temperature	°R
2	LPC outlet temperature	°R
3	HPC outlet temperature	°R
4	LPT outlet temperature	°R
5	Fan inlet pressure	psia
6	Bypass-duct pressure	psia
7	HPC outlet pressure	psia
8	Physical fan speed	rpm
9	Physical core speed	rpm
10	Engine pressure ratio P50/P2	–
11	HPC outlet static pressure	psia
12	Ratio of fuel flow to Ps30	pps/psia
13	Corrected fan speed	rpm
14	Corrected core speed	rpm
15	Bypass ratio	–
16	Burner fuel–air ratio	–
17	Bleed enthalpy	–
18	Required fan speed	rpm
19	Required fan conversion speed	rpm
20	High-pressure turbines cool air flow	lb/s
21	Low-pressure turbines cool air flow	lb/s

FD001 has only one failure mode and one running condition; these characteristics are convenient for analyzing the degradation of the engine. To compare with the existing research, this paper selects FD001, the first set of monitoring data, to verify the method we proposed. In the training set, FD001 recorded the state monitoring data of 20,632 running cycle samples of 100 engines from normal operation to complete failure, and in the test set, the dataset recorded the state monitoring data of 13,097 running cycle states of 100 engines that were terminated at some point before the failure occurred. The purpose of this paper is to predict the number of remaining running cycles (RULs) of the engine before failure in the test set.

### B. Data preprocessing

**Figure 5:**
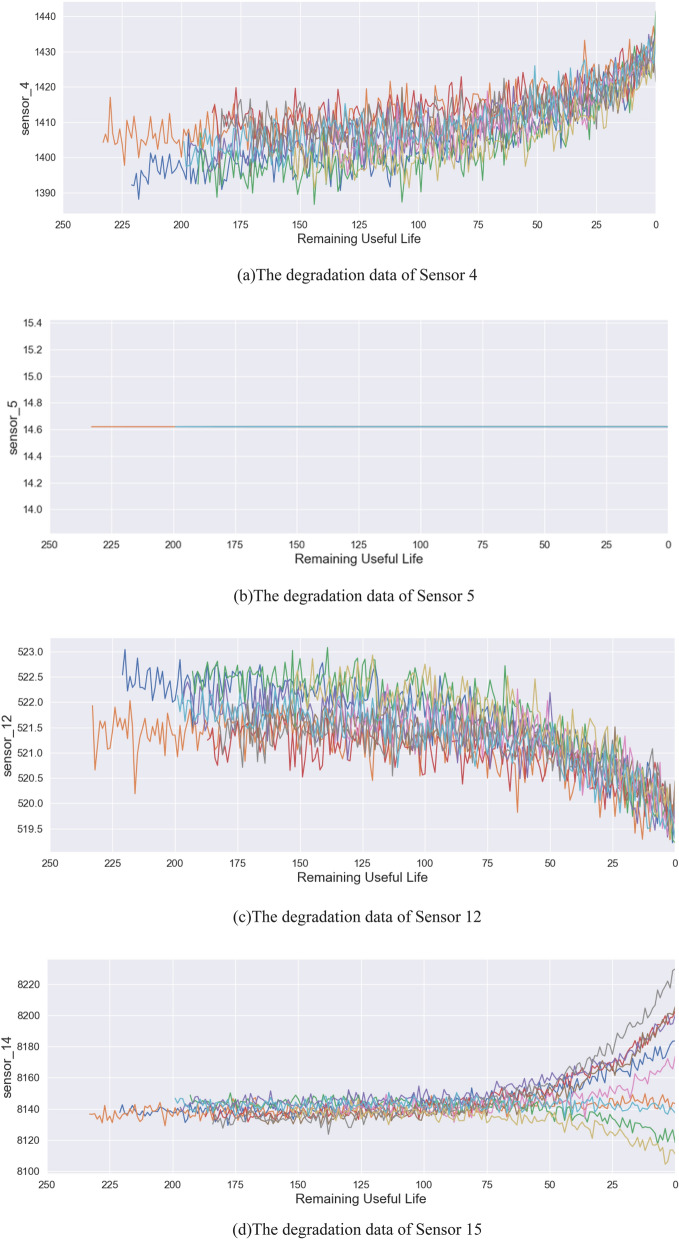
Degradation data of partial sensors.

Because the original data contain considerable random noise, the FD001 dataset needs to be preprocessed before being input into the model; preprocessing ensures the validity of the data and reduces the experimental error. The FD001 data distribution is almost concentrated in the interval of (μ − 3σ, μ + 3σ), and the proportion is 99.73%. The rest of the data exceeding this interval is 0.27%. This part of the data belongs to the gross error and is considered the noise of the original data. In this paper, the 3-sigma criterion is adopted to reduce the noise of the original data. The 3-sigma criterion can remove the monitoring error and avoid the influence of data error on the prediction accuracy; compared with other methods, it has no impact on the original data and ensures the authenticity of the input data. Assuming that multiple measurement data have the same accuracy and distribution, the gross error in the measurement data can be eliminated by using the 3-sigma criterion. The 3-sigma criterion is expressed in Eq. (14).

14 $$ P(\mu - 3\sigma < x < \mu + 3\sigma ) = 0.9973 \, $$

where $\mu$ is the average value of the sample and $\sigma$ represents the standard deviation. The monitoring of the turbine engine is completed by the cooperation of multiple sensors, and the monitoring data of the sensors directly reflect the degradation of the turbine engine. Therefore, we perform denoising on 21 sensor signals of all engine operating cycles in the dataset. The monitoring data on each running cycle in the dataset conform to a Gaussian distribution. As mentioned above, 3-sigma is used for denoising, and multiple data samplings are performed under the same conditions to eliminate the error and noise of each group of monitoring data. After denoising, the trend of sensor monitoring data in all engine life cycles is analyzed, and the degradation data are obtained. According to the experimental data analysis, the change trend of the sensor signal with the engine degradation can be divided into four types: monotonic rise, monotonic decline, irregular change and constant value. It is shown in Fig. 5.

Since some sensor signals are constant during degradation, they do not provide useful information for prediction. Therefore, we delete such data during the data processing stage, and to avoid affecting the reliability of the prediction results, we clean up and reorganize the FD001 training and test sets. Only 14 types of sensor signals are retained to form a new training set and a new test set. Table 2 summarizes the sensor trend information.

**Table 2:** Sensor trends summary.

Trend	Sensor number
Increasing	[2, 3, 4, 8, 11, 13, 14, 15, 17
Decreasing	[7, 12, 20, 21]
Irregular	[9, 14]
unchanged	[1, 5, 6, 10, 16, 18, 19]

For the new FD001 dataset, to eliminate the dimensional influence between sensors and improve the convergence speed of the model, we use the normalization method to process the original monitoring data of the new dataset and limit the data size to the range of [0, 1].

15 $$ X^{*} = \frac{{X - X_{\min } }}{{X_{\max } - X_{\min } }} \, $$

In Eq. (15), $X^{*}$ is the value of each sensor after the normalization at each running cycle of the engine; $X$ is the original sensor data before $X^{*}$ is processed; and $X_{\max }$ and $X_{\min }$ are the maximum and minimum, respectively, of each sensor in each engine running cycle.

### C. Parameter settings

The performance of the model proposed in this paper is greatly affected by the parameters during training. To achieve the best performance, we need to select the best combination of parameters to improve the robustness of the model; the parameter settings mainly include those of the imSSAE and imESN. The fivefold cross-validation method is applied to train models with different parameters. The structure of the imSSAE is 14-8-4-1, which means that the input nodes are 14 (the 14-dimensional sensor data value of each running cycle), the nodes in the two hidden layers are 8 and 4, the output node is 1, and the output eigenvalue is adopted to construct the HI curve. In the imESN, we employ a particle swarm optimization algorithm to optimize the parameters and apply fivefold cross-validation for verification, and each cross-validation is run twice to alleviate the problem of overfitting. In this algorithm, the learning factors $c_{1}$ and $c_{2}$ are both 1.49, the inertia factor $\sigma$ is 0.8, the population size is 200, and the maximum number of iterations *G* is 50. Additionally, this paper uses the RMSE value of the model as an evaluation index to adjust the parameters and improve the prediction effect. The two model parameter combination settings are shown in Table 3.

**Table 3:** Model optimal parameter settings.

imSSAE parameters	Value	imESN parameters	Value
Sparsity parameter $\rho$	0.05	Reservoir node $m$	300
Learning rate	0.01	Spectral radius $\rho (\hat{W})$	0.9
Batch size	64	Sparsity SD	0.05
Epoch	50	Leakage rate $\lambda$	0.23
Dropout	0.2	Scaling factor IS	0.4
Network structure	14-8-4-1		

### D. Experiments result analysis

**Figure 6:**
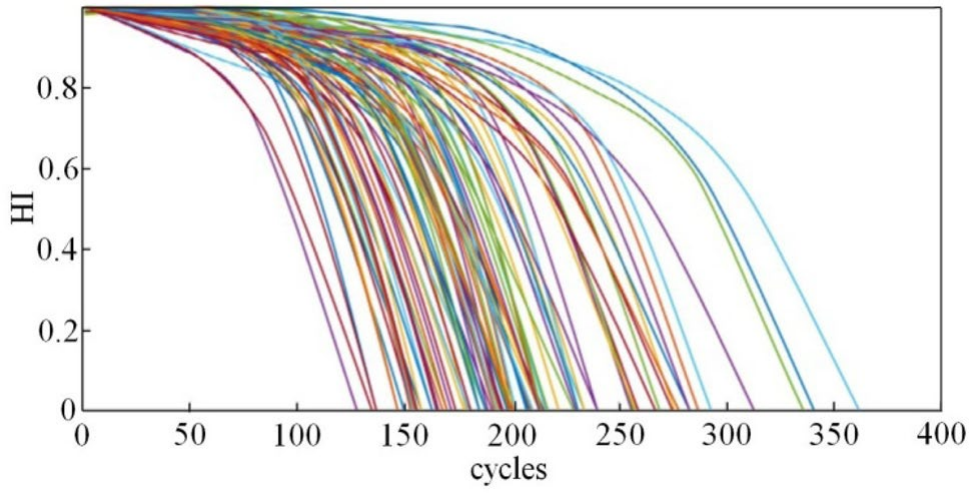
HI curve of the 100 engines.

**Figure 7:**
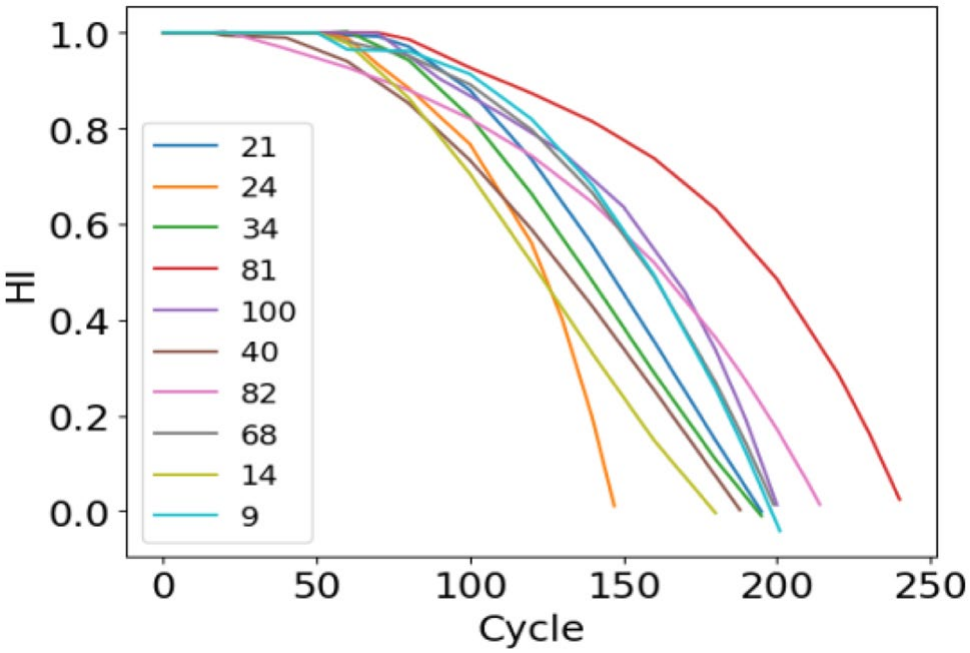
10 HI curve of the training set.

**Figure 8:**
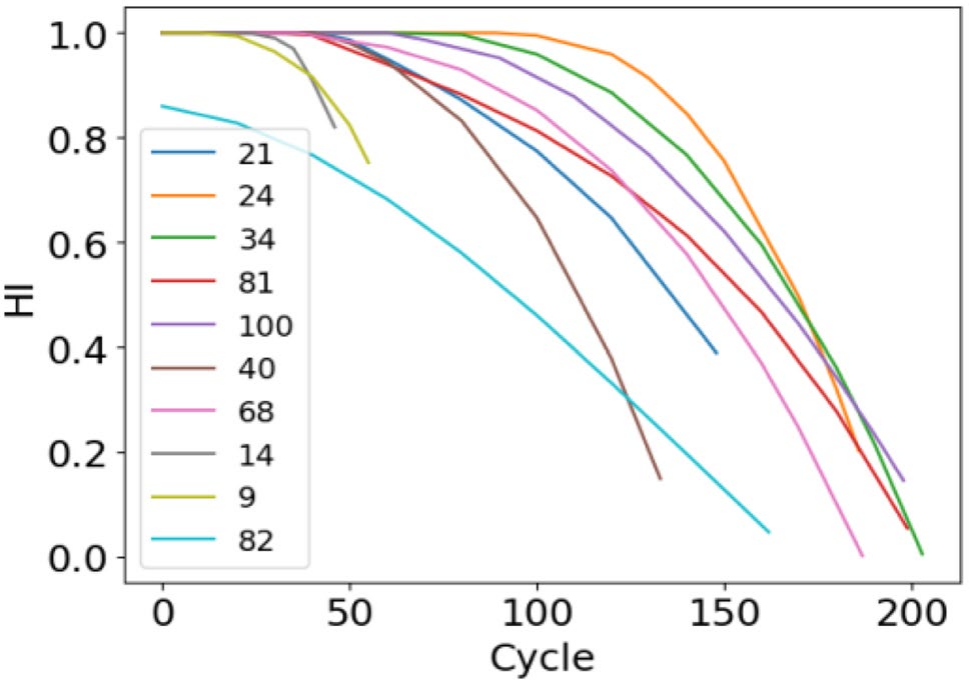
10 HI curve of the test set.

During training, we input the 14-dimensional sensor data of each running cycle into the encoder of the imSSAE for feature extraction so that each running cycle of the engine obtains one feature. Then, the HI value of each running cycle is constructed according to the features, and finally, the HI curve of each engine is obtained. Figure 6 depicts the HI curve of 100 engines. To ensure that the characteristics of the original HI curve remain unchanged, fitting and smoothing are performed. We randomly choose 10 from 100 engines for the experiment, and the curves of the HI training set and test set are shown in Figs. 7 and 8.

As depicted in the above figures, the HI curve can well describe the degradation process of the engine and has good monotonicity. To better measure the construction effect of the HI curve, the HI curve evaluation index is used to compare the commonly used method of multisensor degradation state modeling proposed in references^19,20^ and the method proposed in this paper. The results are shown in Table 4. The values are the average index of 100 HI curves and 100 engines. Table 4 shows that the method proposed in this paper is superior to other methods in terms of time relevance and monotonicity mainly because our method performs noise reduction and reorganization of the dataset before data processing, thereby leading to better monotonicity and little interference. In contrast, PCA and ELM_AE have monotonic inconsistencies due to noise interference, thus resulting in monotonic reduction. The high time correlation indicates that the ability to capture potential degraded features is strong. In summary, the feature extraction and HI construction of the method proposed in this paper can better reflect the engine's degradation process.

**Table 4:** Index evaluation results.

Method	$Mon_{avg}$	$Corr_{avg}$
Linear regression	0.8011	0.6508
BP	0.5399	0.6567
DBN	0.7422	0.7273
PCA	0.054	0.516
ELM_AE	0.049	0.426
SDAE	0.428	0.825
The proposed method	0.824	0.837

**Figure 9:**
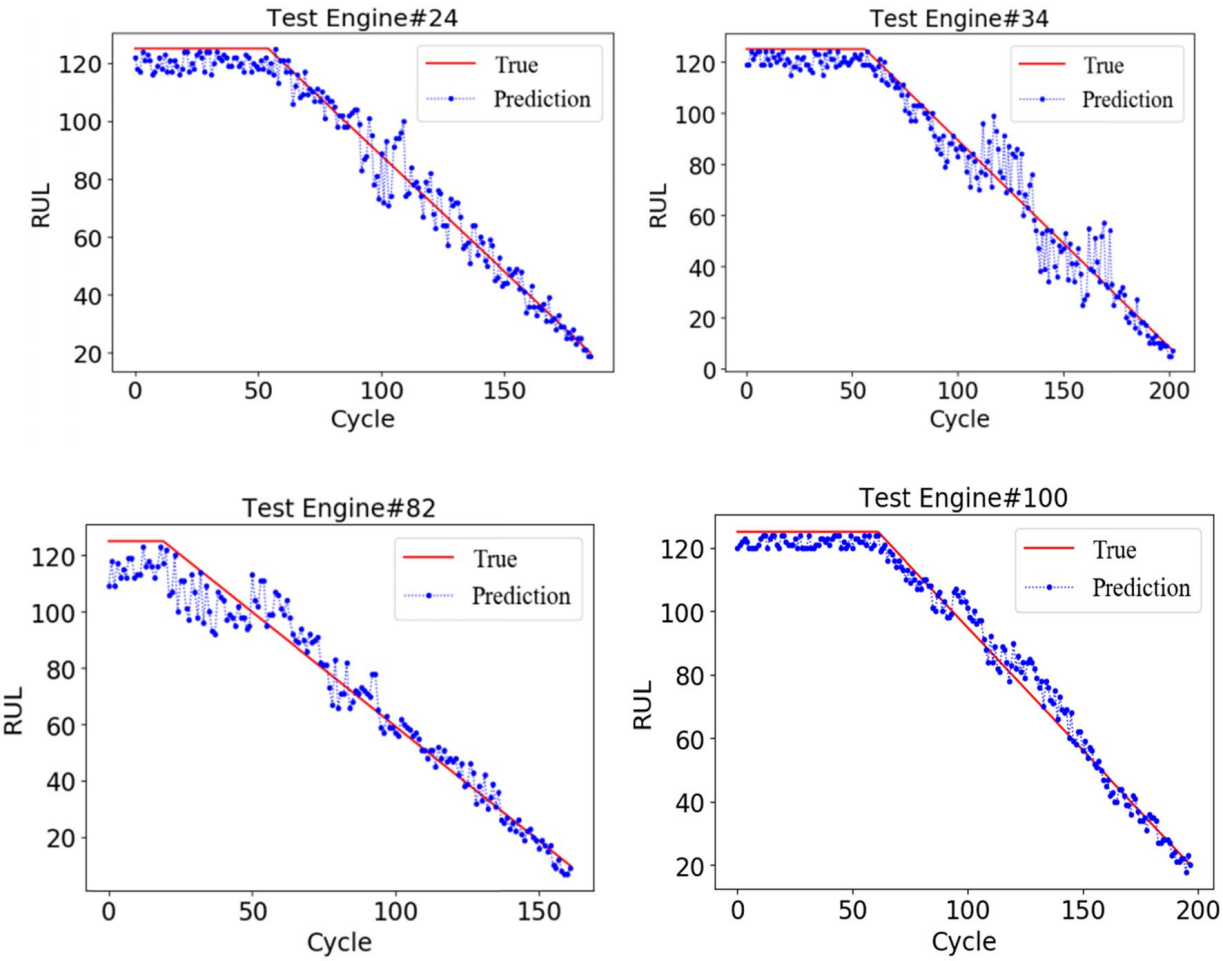
RUL prediction results of four monitoring units.

Referring to the current popular literature^21,22^, to analyze the RUL of each engine unit, a piecewise linear degradation model is proposed to determine the target RUL in this paper. The operation state of the engine can be considered healthy in the initial stage, and the degradation will be obvious after a period of runs or after the engine is used to a certain degree; that is to say, the normal working state of the engine is a constant value, and after a certain period, the RUL of the engine will linearly decrease. In this paper, the initial constant RUL value of the observation data in the degradation stage is 125, and the RUL prediction results of the four monitoring units (24, 34, 82, and 100) are randomly selected, as shown in Fig. 9. The figure shows that the early RUL prediction is close to a constant value, thus indicating that the engine is in a healthy state, and as the running cycle increases, the fault emerges. The prediction result basically shows a linear decline until failure occurs, and some engines (such as 82) have a poor initial prediction effect due to the insignificant initial degradation trend. As the cycle increases, the prediction error decreases. The prediction effect of the engine is best when it is close to failure, thus showing that the features at this time are the most prominent.

**Figure 10:**
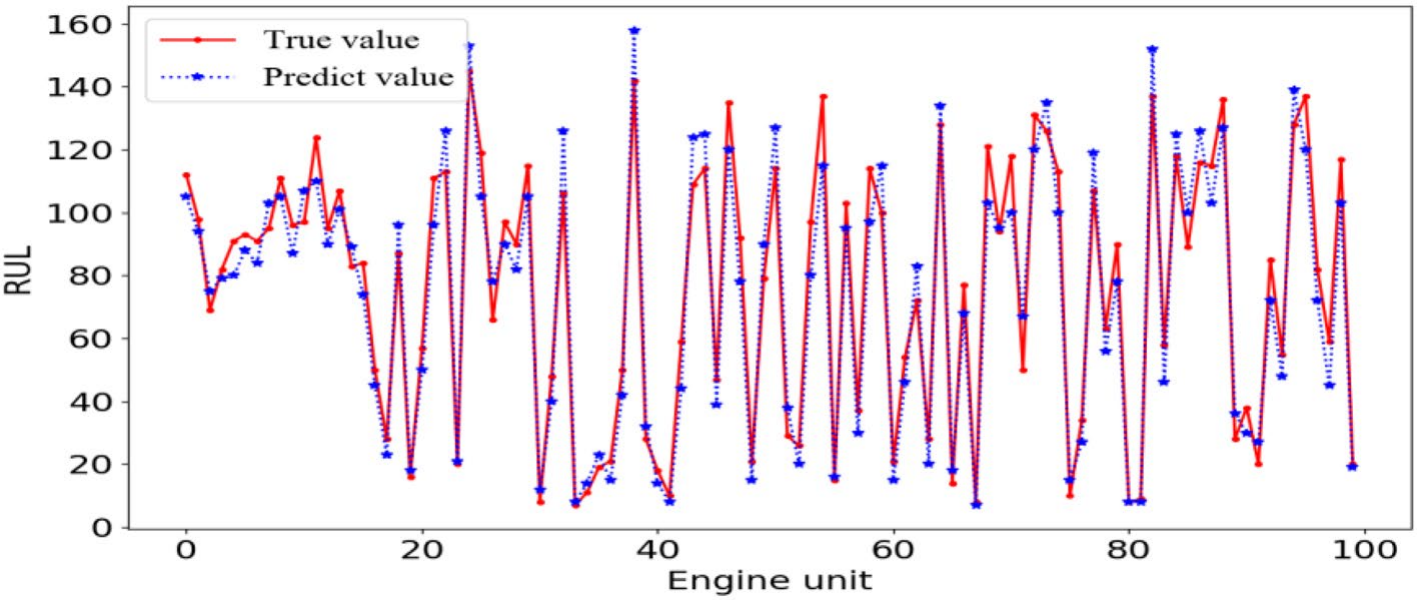
The RUL prediction results of the test set.

Figure 10 shows the comparison results of the true RUL and the predicted RUL of each engine in the test set. To measure the prediction performance of the model more comprehensively, we select the latest and popular life prediction method to compare the evaluation indices of our method and other methods on the same dataset. These methods are evaluated based on RMSE and score, the calculation formula is show in Eq. (16) and Eq. (17). In order to enhance comparability, the same evaluation index is adopted in this paper; Experiments show that SAE extracts features and maps them from high dimension to low dimension. If the dimension gap is too large and the hidden features are inaccurate, the experimental results are not ideal, however, imSSAE can extract the features layer by layer in the form of stacking, improve the training efficiency, and the final hidden features can better express the original data. ESN without attention mechanism cannot adaptively process different features, and cannot process the data set in this paper. ESN with attention mechanism can adaptively process features, and optimize and update the internal parameters of ESN network, which can make the internal parameters of the network reach an optimal state and improve the prediction accuracy. In addition, the proposed method is also compared with others; the results are shown in Table 5.

**Table 5:** results of various mainstream RUL prediction methods.

Model	RMSE	Score
The proposed method	10.14	197
HDNN^23^	13.02	245
DCNN^24^	12.61	274
LSTM-FNN^24^	16.14	338
DBN^25^	15.21	418
MLP^26^	16.78	560
SVM^26^	40.72	7703
RF^26^	17.91	480
Autoencoder-BLSTM^27^	13.63	261
VAE-D2GAN^28^	11.60	221
CNN-LSTM^29^	14.40	290

Root mean square error (RMSE): this measurement standard is used to measure the deviation between the predicted value and the real value. It is a common evaluation index, which provides a consistent prediction weight for the front and rear periods of turbofan engine. The calculation formula is as follows:

16 $$ RMSE = \sqrt {\frac{1}{n}\sum\nolimits_{i = 1}^{n} {(y_{i} - \hat{y}_{i} )}^{2} } $$

where $\mathrm{n}$ is the sample number of turbofan engine test data; ${\mathrm{y}}_{\mathrm{i}}$ refers to the true value of RUL of the $\mathrm{i}$th turbofan engine, ${\widehat{\mathrm{y}}}_{\mathrm{i}}$ is the predicted value of RUL of the $\mathrm{i}$th turbofan engine.

Scoring function (SF): RMSE has the same punishment for early prediction and later prediction in terms of prediction. Different from RMSE, the scoring function is more inclined to early prediction (RUL predicted value is less than the actual value) than later prediction. The scoring function applies larger punishment for overestimated value and smaller punishment for underestimated value. The scoring function is calculated as follows:

17 $$ SF = \left\{ {\begin{array}{*{20}c} {\sum\nolimits_{i = 1}^{n} {(e^{{ - (\frac{{y_{i} - \hat{y}_{i} }}{13})}} - 1),y_{i} - \hat{y}_{i} < 0;} } \\ {\sum\nolimits_{i = 1}^{n} {(e^{{(\frac{{y_{i} - \hat{y}_{i} }}{10})}} - 1),y_{i} - \hat{y}_{i} \ge 0;} } \\ \end{array} } \right. $$

where SF represents the score value and $n$ is the number of samples. The lower the value of SF, the better the effect of the model.

Compared with other methods, the method proposed in this paper has the minimum of the two scoring indicators, namely, RMSE and score; specifically, the RMSE obtained by the proposed method is 39.5–75% lower than that obtained by traditional machine learning methods, such as the MLP, SVM, and RF; and the score obtained by the proposed method is 59% lower than that obtained by the RF. The method proposed in this paper obtains these results because the neural network structure of the method can adaptively extract different features from engines and optimize the network parameters to improve the prediction effect of the model. Compared with the RMSE and score obtained by the hybrid neural network structure (such as the DBN, CNN-LSTM, and HDNN), the RMSE and the score obtained by the proposed method is reduced by 22.1–33.3% and 19.5–52.8%, respectively, because the encoder of the imSSAE can obtain low-dimensional and more effective features and the imESN can reasonably construct the HI curve to improve the prediction effect. Compared with the RMSE and the score obtained by the autoencoder-BLSTM, the RMSE and score obtained by the proposed method is reduced by 12.5% and 25.6%, respectively; and compared with the RMSE and the score obtained by the VAE-D2GAN with encoder structure, the RMSE and score obtained by the proposed method is reduced by 10.8% and 24.5%, respectively. These results show that the data preprocessing used in this paper before feature extraction is more advantageous in summary, the prediction accuracy of this method on the C-MAPSS dataset is improved, and this method can be used to predict the maintenance time of the turbofan engines without expert knowledge and physical knowledge.

## Conclusions

This study is intended to present an efficient and accurate method for RUL prognosis of turbofan engine. In this work, a deep feature extraction based method is proposed to automatically extract features with little prior knowledge and construct HI curve, additionally, facing the phenomenon of different feature information existing in the input data, an attention mechanism was presented to improve the performance of the RUL prognosis, a turbofan engine data set FD001 was adopted to demonstrate the effectiveness of the proposed methodology. Compared with other methods, the proposed method has obvious advantages in prediction accuracy. However, there is still room for improvement in predicting the remaining useful of turbofan engine, and further research is still needed. In this study, it has been assumed that samples in the data set are balanced; imbalanced sample will affect the prediction effect, the current approach shall be modified to consider the imbalance in data set. In addition, the failure threshold in the experiment is given in the data set, how to adaptively calculate the failure threshold according to different data sets is the subject of a new research.
